# The Microwave-Assisted Green Synthesis of TiC Powders

**DOI:** 10.3390/ma9110904

**Published:** 2016-11-08

**Authors:** Hui Wang, Wencheng Zhu, Yanchun Liu, Lingke Zeng, Luyi Sun

**Affiliations:** 1 College of Materials Science & Engineering, South China University of Technology, Guangzhou 510640, China; 18814123136@163.com (W.Z.); lingke@scut.edu.cn (L.Z.); 2 Department of Chemical & Biomolecular Engineering and Polymer Program, Institute of Materials Science, University of Connecticut, Storrs, CT 06269, USA; 3 Guangzhou Redsun Gas Appliances Co., Ltd., Guangzhou 510460, China; lyc21@163.com

**Keywords:** TiC powders, microwave-assisted synsthesis

## Abstract

Titanium carbide (TiC) is an important engineering material and has found widespread applications. Currently, TiC is typically synthesized through carbothermal reduction, requiring a high temperature (ca. 1700–2300 °C) and long reaction time (ca. 10–20 h), which is not eco-friendly. During a conventional reaction path, anatase TiO_2_ (A-TiO_2_) was first converted to rutile TiO_2_ (R-TiO_2_), which was subsequently reduced to TiC. Herein, we explored the synthesis of TiC powders with the assistance of microwave heating. In particular, we achieved the conversion of A-TiO_2_, which was more reactive than R-TiO_2_ for the carbothermal reduction, to TiC, which was directly due to quick microwave heating. As such, the carbothermal reduction started at a much lower temperature of ca. 1200 °C and finished within 30 min when reacting at 1400 °C, leading to significant energy saving. This study shows that microwave-assisted synthesis can be an effective and green process for preparing TiC powders, which is promising for future large-scale production. The influence of the reaction temperature, the reaction duration, and the carbon content on the synthesis of TiC powders was investigated.

## 1. Introduction

Titanium carbide (TiC) is an important engineering material because of its superior hardness, high melting temperature, good electrical conductivity, and outstanding abrasion resistance [1,2,3]. Therefore, TiC powders have been used extensively in cutting tools, grinding wheels, polishing pastes, etc. [3,4,5,6].

Currently, TiC powders are mainly synthesized by the carbothermal reduction of TiO_2_, and the heat during the process of reaction is usually provided by an external heating system. The synthesis is typically in the temperature range of 1700–2300 °C for 10–20 h [7]. The size of the synthesized TiC powders not only depends on the size of raw materials, but also on the synthesis conditions. Therefore, it is difficult to prepare uniform TiC powders by conventional heating processes [8,9].

As an alternative heating technology, microwave heating has advantages such as high thermal efficiency, selective heating, quick heating, and short processing time. It has been implemented in many industrial applications in recent years [10,11,12]. The major difference between microwave heating and conventional heating is the inverse temperature profile inside microwave-heated samples [13,14]. The center of the sample becomes hotter than the surface, which is exposed to the colder furnace atmosphere [15]. Many materials including ceramics, polymers, and metallic powders can be directly exposed to microwaves [16]. In microwave carbothermal reduction process, two factors might be favorable for lowering the onset reaction temperature. One is the existence of thermal and non-thermal effects from microwave heating [17]. The other is the uniform heating achieved by the energy directly delivered to the starting materials via molecular-level interactions under an electromagnetic field [18,19]. Microwave-assisted synthesis is attractive for the preparation of TiC because it is faster and more effective than conventional heating. Carbon, one of the reactants for the synthesis of TiC, is a very effective absorber of microwave radiation, offering extra facilitation of the reaction. Cross and coworkers [20,21] made some early explorations of the synthesis of TiC via microwave heating and proved that it was viable and indeed more effective to synthesize TiC via microwave heating.

In this report, microwave heating was adopted to synthesize TiC powders by carbothermal reduction of TiO_2_. The influence of reaction temperature and carbon content on the phase composition of the produced TiC powders was systematically investigated. In particular, we aimed to achieve the direct conversion of anatase TiO_2_ (A-TiO_2_), which was more reactive than rutile TiO_2_ (R-TiO_2_), for the carbothermal reduction to TiC by taking advantage of quick microwave heating.

## 2. Experimental

### 2.1. Materials

Both A-TiO_2_ powders (99%, ~40 nm in diameter) and carbon black (99%, ~10 nm in diameter) were purchased from Tuoyi Co. (Guangzhou, China). Ethanol was acquired from Fuyu Fine Chemical Co. (Fuzhou, China). Polyacrylic acid (PAA) was obtained from KeMiOU Chemical Co. (Tianjin, China). All the chemicals were used as received without further purification.

### 2.2. Synthesis

First, a 20.0 wt % A-TiO_2_ ethanol dispersion was ultrasonicated for 30 min to form a uniform system, which was mixed with polyacrylic acid (PAA/TiO_2_ = 0.5 wt %) to stabilize the dispersion of A-TiO_2_ in ethanol. Subsequently, a pre-determined amount of carbon black was mixed with the above dispersion. The reaction precursor was obtained after evaporating ethanol.

The synthesis was carried out in a KL-2D-16 microwave furnace (Kailin Microwave Equipment Co., Guangzhou, China). During reaction, the microwave frequency was 2450 ± 50 MHz, and microwave power was 6000 W. Thermocouples were used to measure the temperatures during reaction. The entire reaction was carried out under argon protection.

The carbothermal reduction of TiO_2_ is a complex process. Under a conventional heating process, before the reduction reaction, A-TiO_2_ is first converted to R-TiO_2_. With the rising of temperature, the reduction consists of a series of intermediate reactions, producing various intermediate products and phases (Equations (1)–(4)). The general procedures of the carbothermal reduction reaction between carbon black and R-TiO_2_ through conventional heating to generate TiC are as follows [22,23]:

(1) $$2R-{\mathrm{TiO}}_{2}+C={\mathrm{Ti}}_{2}O_{3}+\mathrm{CO};$$

(2) $${\mathrm{Ti}}_{2}O_{3}+C=2\mathrm{TiO}+\mathrm{CO};$$

(3) TiO+2C=TiC+CO.

Total reaction:

(4) $${\mathrm{TiO}}_{2}+3C=\mathrm{TiC}+2\mathrm{CO}.$$

**Figure 1:**
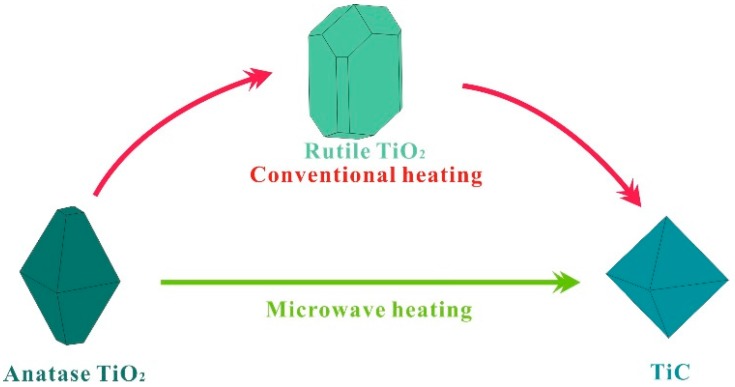
Schematic of different reaction routes between conventional and microwave heating.

In this work, we aimed to achieve a direct reduction of A-TiO_2_ to TiC under the assistance of microwave heating, as illustrated in Figure 1. Such a direction reduction route is expected to occur at a lower temperature, thus saving energy and time. In order to investigate the reduction process of TiO_2_ by microwave heating, we monitored the phase transition of the reaction products at various temperatures and reaction durations by X-ray diffraction, and studied the influence of carbon content on the synthesis of the TiC powders.

### 2.3. Characterization

Phase composition of the reaction products was determined by X-ray diffraction on a PANalytical X-ray diffractometer (XRD, monochromated Cu Kα radiation) at 25 °C. The morphology of the powders was imaged by scanning electron microscopy (SEM) on a Nova S-430 microscope operated at 20 kV, which was equipped with an energy-dispersive X-ray spectrometer (EDS).

## 3. Results and Discussion

### 3.1. Effect of Temperature and Reaction Time

**Figure 2:**
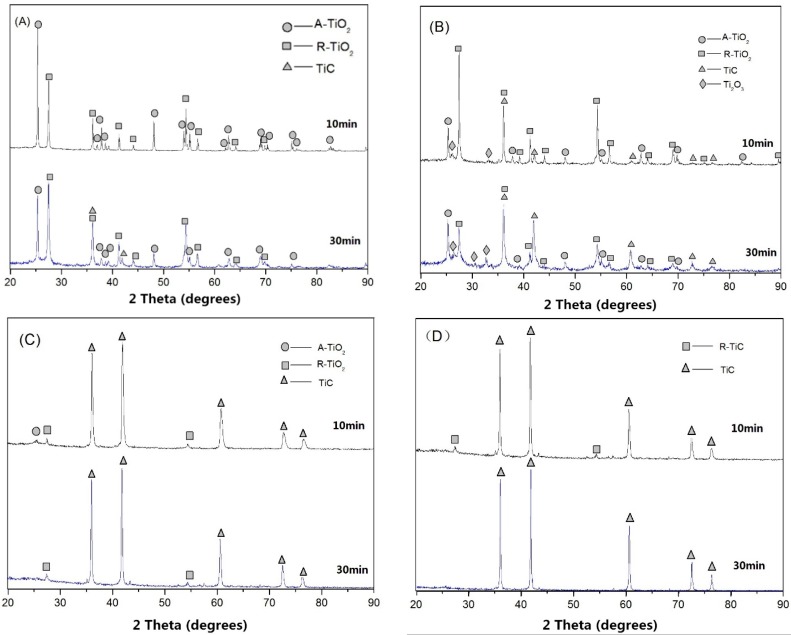
X-ray diffractometer (XRD) patterns of the synthesized samples at various temperatures: (**A**) 1100 °C; (**B**) 1200 °C; (**C**) 1300 °C; (**D**) 1400 °C.

The XRD patterns of the samples synthesized at various temperatures (1100, 1200, 1300, and 1400 °C) and durations of reaction (10 and 30 min) at a TiO_2_ and C molar ratio of 1:3.6 are presented in Figure 2.

Figure 2A displays the XRD patterns of the samples reacted at 1100 °C for 10 and 30 min. It shows that TiC phase was not formed at 1100 °C for 10 min of reaction, but a small amount of TiC was generated after 30 min of reaction. The conversion from A-TiO_2_ to R-TiO_2_ was observed at this temperature. A number of studies reported that A-TiO_2_ began to transform to R-TiO_2_ at a temperature up to 610 °C [24,25]. With an increase in reaction time, more R-TiO_2_ was generated, as evidenced by stronger R-TiO_2_ phase diffraction peaks in the XRD pattern. The XRD patterns of the samples synthesized at 1200 °C (Figure 2B) showed that most A-TiO_2_ was converted to be R-TiO_2_; meanwhile, Ti_2_O_3_ started to form after 10 min of reaction at 1200 °C. After 30 min of reaction at 1200 °C, the TiC phase started to appear as evidenced by the corresponding XRD peaks. The TiC phase could be clearly observed on the XRD pattern when the reaction temperature was raised to 1300 °C. With an increase in reaction time, a higher concentration of TiC was generated, as supported by the more intensive diffraction peaks of TiC (Figure 2C). According to the XRD pattern, the virtually pure TiC phase was synthesized after 30 min of reaction at 1400 °C (Figure 2D).

Based on the above observations, the reaction mechanism is proposed as follows. At 1100 °C, the main reaction was the transformation from A-TiO_2_ to R-TiO_2_, but this is a relatively slow process:

(5) $$A-{\mathrm{TiO}}_{2}\to R-{\mathrm{TiO}}_{2}.$$

It was reported that the activity of A-TiO_2_ with C was higher than that of R-TiO_2_ [26,27,28], so the rate of Reaction (6) is faster than that of Reaction (1):

(6) $$2A-{\mathrm{TiO}}_{2}+C\to {\mathrm{Ti}}_{2}O_{3}\left(s\right)+\mathrm{CO}\left(g\right).$$

During conventional heating, A-TiO_2_ tends to transform to R-TiO_2_ before the reduction reaction. This is one of the key reasons that a much higher temperature; thus, much more energy is required to convert the less active R-TiO_2_ to TiC. If one can quick heat to directly convert A-TiO_2_ to TiC, it is much more favorable in terms of energy consumption. Microwave heating can help achieve this process. Because the rate of microwave heating is very fast, it takes a very short time to raise the temperature from 1100 to 1200 °C. Therefore, when the temperature was quickly increased to 1200 °C, some A-TiO_2_ remained. Such unconverted A-TiO_2_ can quickly react with C to form TiC at relatively lower temperatures compared with the reduction reaction temperature of R-TiO_2_ to TiC. Figure 2C shows that, after 30 min of reaction at 1300 °C, there was still some R-TiO_2_ left but no A-TiO_2_. As such, a high heating rate is very desirable to directly reduce A-TiO_2_ to TiC, which allows for the synthesis of TiC at lower temperatures while saving energy. After 30 min of reaction at 1300 °C, most TiO_2_ was reacted to form TiC, which began to be the dominating phase.

**Figure 3:**
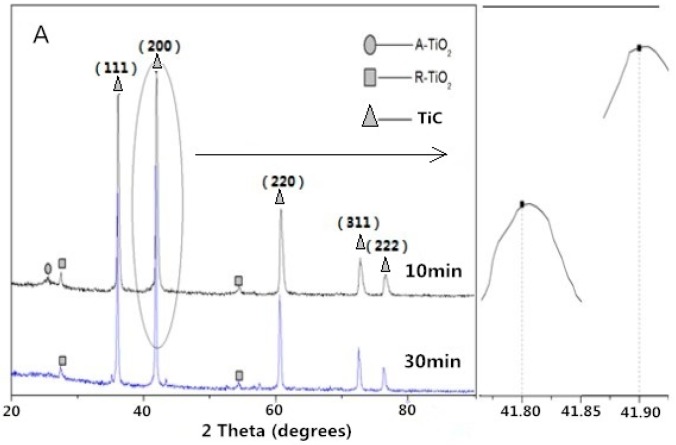

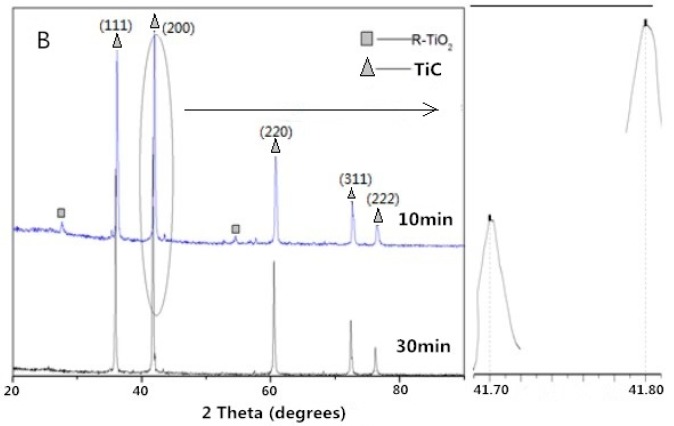
XRD patterns of the samples synthesized at (**A**) 1300 °C and (**B**) 1400 °C.

With an increase in reaction temperature and time, it was observed that the (*200*) peak of the synthesized TiC shifted from 41.90° to 41.70° (Figure 3A,B). The lattice constant of the TiC (1300 °C, 10 min) and TiC (1400 °C, 30 min) were calculated to be 4.321 Å and 4.324 Å, lower than the standard value of 4.327 Å. The results show that a higher temperature and longer reaction time are beneficial for the growth of TiC, which was also reported by Preiss et al. [29,30].

**Figure 4:**
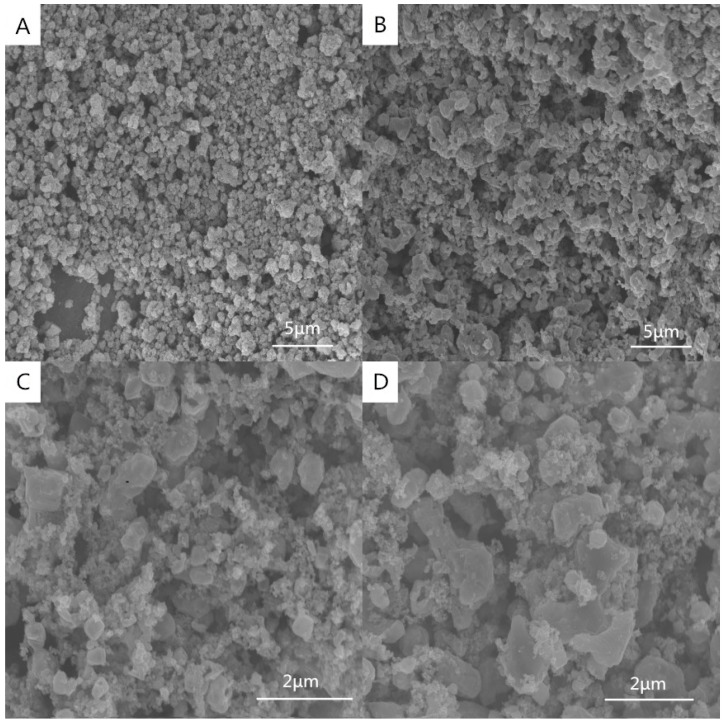
SEM images of the products synthesized at various temperatures for 30 min: (**A**) 1100 °C; (**B**) 1200 °C; (**C**) 1300 °C; (**D**) 1400 °C.

SEM images of the samples synthesized at various temperatures (1100, 1200, 1300, and 1400 °C) for 30 min are shown in Figure 4. With an increase in reaction temperature, the size of the powders became larger, changing from ca. 0.2 μm at 1100 °C to ca. 0.4 μm at 1200 °C. When the reaction temperature was increased to 1300 °C, the TiC powders exhibited a pseudocubic morphology (Figure 4C) with a particle size of ca. 0.7 μm. It should be noted that, although no other diffraction peaks were observed in Figure 2D, amorphous carbon should still exist because excessive carbon was added to ensure a complete carbothermal reduction, because carbon black acts as both the carbon source and the media to transform microwave dielectric to heat.

### 3.2. Effect of Carbon Content

TiO_2_ is a poor microwave absorber, while carbon black absorbs microwave very effectively. During the reaction, carbon black not only participates in the reaction but also serves as a medium to absorb microwave radiation to help heat the mixture of the reactants. Its content was reduced along the reaction; therefore, excessive carbon black was added during the reactions. In order to study the role of carbon content in the process of the reduction of TiO_2_, mixtures of TiO_2_ and C at various molar ratios (1:3.0; 1:3.2; 1:3.4, and 1:3.6) were reacted at 1400 °C for 30 min via microwave heating.

Table 1 presents the results calculated from the data of the XRD and EDS measurements of the products from the samples starting at various TiO_2_ and C mole ratios. The results showed that, with an increasing amount of carbon in the mixture, both the concentration and lattice constant of TiC increased, which suggests that a higher concentration of C is favorable for the growth of TiC.

## 4. Conclusions

In conclusion, our experimental results showed that pure and pseudocubic TiC phase could be synthesized by directly reducing A-TiO_2_ instead of R-TiO_2_ with the assistance of quick microwave heating. The lattice constant of TiC increases with an increasing reaction temperature and time. Carbon black acts as both the carbon source and the media to transform microwave dielectric to heat. A high ratio of C to TiO_2_ in the starting materials is favorable for improving the conversion rate and quality of TiC powders. Compared with the carbothermal reduction using conventional heating to synthesize TiC powders, which typically requires a temperature of 1700–2400 °C for 10 to 24 h, TiC powders could be synthesized at a temperature of as low as 1200 °C during microwave heating, and the carbothermal reduction can be finished within 30 min at 1400 °C. Therefore, the preparation of TiC powders via microwave heating is much more energy-effective and thus promising for large-scale production.

## Figures and Tables

**Table 1:** Product composition after reaction at 1400 °C for 30 min under various TiO_2_ and C ratios.

TiO_2_:C (mol:mol/wt:wt)	Composition of Product (wt %)			TiC (*200*) Peak Position (Degree)	Lattice Constant of TiC (Å)
	TiO_2_	C	TiC		
1:3.0/1:0.45	15.5	4.2	80.3	41.92	4.319
1:3.2/1:0.48	6.7	5.7	87.6	41.86	4.320
1:3.4/1:0.51	2.4	8.3	89.3	41.78	4.322
1:3.6/1:0.54	0	9.4	90.6	41.70	4.324

## References

[1] Shin Y., Li X.S., Wang C., Coleman J.R., Exarhos G.J. (2004). Synthesis of hierarchical titanium carbide from titania-coated cellulose paper. Adv. Mater..

[2] Pierson H.O. (1996). Handbook of Refractory Carbides & Nitrides: Properties, Characteristics, Processing and Apps.

[3] Toth L. (2014). Transition Metal Carbides and Nitrides.

[4] Koc R. (1998). Kinetics and phase evolution during carbothermal synthesis of titanium carbide from ultrafine titania/carbon mixture. J. Mater. Sci..

[5] El-Eskandarany M.S. (2000). Structure and properties of nanocrystalline TiC full-density bulk alloy consolidated from mechanically reacted powders. J. Alloys Compd..

[6] Ushakov A., Karpov I., Lepeshev A., Krushenko G. (2011). Physicochemical properties of nanomodifiers based on electric arc titanium nitride powder for polymer nanocomposite materials. Tekhnol. Met..

[7] Kappe C., Dallinger D., Murphree S. (2009). Practical Microwave Synthesis for Organic Chemists: Strategies, Instruments, and Protocols.

[8] Choi Y., Rhee S.-W. (1993). Effect of aluminium addition on the combustion reaction of titanium and carbon to form TiC. J. Mater. Sci..

[9] Koc R., Folmer J. (1997). Carbothermal synthesis of titanium carbide using ultrafine titania powders. J. Mater. Sci..

[10] Thakur S.K., Kong T.S., Gupta M. (2007). Microwave synthesis and characterization of metastable (Al/Ti) and hybrid (Al/Ti + SiC) composites. Mater. Sci. Eng. A.

[11] Rajkumar K., Aravindan S. (2009). Microwave sintering of copper-graphite composites. J. Mater. Process. Technol..

[12] Leparoux S., Vaucher S., Beffort O. (2003). Assessment of microwave heating for sintering of Al/SiC and for in-situ synthesis of TiC. Adv. Eng. Mater..

[13] Campanone L., Zaritzky N. (2005). Mathematical analysis of microwave heating process. J. Food Eng..

[14] Ciacci T., Galgano A., Di Blasi C. (2010). Numerical simulation of the electromagnetic field and the heat and mass transfer processes during microwave-induced pyrolysis of a wood block. Chem. Eng. Sci..

[15] Rosa R., Veronesi P., Leonelli C. (2013). A review on combustion synthesis intensification by means of microwave energy. Chem. Eng. Process. Process Intensif..

[16] Mishra R.R., Sharma A.K. (2016). Microwave–material interaction phenomena: Heating mechanisms, challenges and opportunities in material processing. Compos. A Appl. Sci. Manuf..

[17] Porcelli M., Cacciapuoti G., Fusco S., Massa R., d’Ambrosio G., Bertoldo C., De Rosa M., Zappia V. (1997). Non-thermal effects of microwaves on proteins: Thermophilic enzymes as model system. FEBS Lett..

[18] Moshtaghioun B., Poyato R., Cumbrera F., de Bernardi-Martin S., Monshi A., Abbasi M., Karimzadeh F., Dominguez-Rodriguez A. (2012). Rapid carbothermic synthesis of silicon carbide nano powders by using microwave heating. J. Eur. Ceram. Soc..

[19] Zhang H., Li F., Jia Q., Ye G. (2008). Preparation of titanium carbide powders by sol-gel and microwave carbothermal reduction methods at low temperature. J. Sol-Gel Sci. Technol..

[20] Binner J.G.P., Hassine N.A., Cross T.E. (1995). The possible role of the pre-exponential factor in explaining the increased reaction rates observed during the microwave synthesis of titanium carbide. J. Mater. Sci..

[21] Hassine N.A., Binner J.G.P., Cross T.E. (1995). Synthesis of refractory metal carbide powders via microwave carbothermal reduction. Int. J. Refract. Met. Hard Mater..

[22] Holt J., Munir Z. (1986). Combustion synthesis of titanium carbide: Theory and experiment. J. Mater. Sci..

[23] Zeng L.K., Liu Y.C., Zhu W.C., Liu P.A., Wang H., Cheng X.S., Liang Q.Y. (2014). Investigation on the continuous microwave synthesis of nano titanium carbide powder. Adv. Mater. Res..

[24] Chen Y.-F., Lee C.-Y., Yeng M.-Y., Chiu H.-T. (2003). The effect of calcination temperature on the crystallinity of TiO_2_ nanopowders. J. Cryst. Growth.

[25] Colmenares J., Aramendia M., Marinas A., Marinas J., Urbano F. (2006). Synthesis, characterization and photocatalytic activity of different metal-doped titania systems. Appl. Catal A Gen..

[26] Lucarelli L., Nadtochenko V., Kiwi J. (2000). Environmental photochemistry: Quantitative adsorption and FTIR studies during the TiO_2_-photocatalyzed degradation of orange II. Langmuir.

[27] Diamandescu L., Vasiliu F., Tarabasanu-Mihaila D., Feder M., Vlaicu A., Teodorescu C., Macovei D., Enculescu I., Parvulescu V., Vasile E. (2008). Structural and photocatalytic properties of iron-and europium-doped TiO_2_ nanoparticles obtained under hydrothermal conditions. Mater. Chem. Phys..

[28] Jung Y.-S., Kim D.-W., Kim Y.-S., Park E.-K., Baeck S.-H. (2008). Synthesis of alumina-titania solid solution by sol-gel method. J. Phys. Chem. Solids.

[29] Preiss H., Berger L.-M., Schultze D. (1999). Studies on the carbothermal preparation of titanium carbide from different gel precursors. J. Eur. Ceram. Soc..

[30] Cao Y., Zhang H., Li F., Lu L., Zhang S. (2015). Preparation and characterization of ultrafine ZrB_2_–SiC composite powders by a combined sol-gel and microwave boro/carbothermal reduction method. Ceram. Int..

